# Genome Sequence of Fowlpox Virus-Integrated Reticuloendotheliosis Virus from a Rio Grande Wild Turkey (Meleagris gallopavo *intermedia*)

**DOI:** 10.1128/mra.00174-22

**Published:** 2022-05-16

**Authors:** Bianca Willis, Camille Trautman, Faith Cox, Tiffany Lujan, Jason Hardin, Robert Dittmar, Camila Romano, Jeff Brady, Dustin Edwards

**Affiliations:** a Department of Biological Sciences, Tarleton State University, Stephenville, Texas, USA; b Texas Parks and Wildlife Department, Buffalo, Texas, USA; c Texas Parks and Wildlife Department, Kerrville, Texas, USA; d Instituto de Medicina Tropical de São Paulo e Hospital das Clínicas da Faculdade de Medicina, Universidade de São Paulo, São Paulo, Brazil; e Texas A&M AgriLife Research, Stephenville, Texas, USA; KU Leuven

## Abstract

We report the genome sequence of a nearly intact reticuloendotheliosis virus (REV) insertion within a field strain of fowlpox virus from a Rio Grande wild turkey in Gillespie County, TX. The proviral REV genome comprises 7,943 bp and contains partial long terminal repeats.

## ANNOUNCEMENT

Fowlpox virus (FWPV) is a double-stranded DNA virus within the family *Poxviridae* that infects multiple avian species, including domestic poultry and wild birds (1). FWPV has two presentations: the cutaneous or dry form, in which lesions form on the nonfeathered areas of the legs, head, and body (2), and the diphtheritic or wet form, in which lesions form in the mucous membranes of the mouth and respiratory tract (2). Several FWPV vaccine and field strains contain integrated partial segments, or near or fully intact genomes, of the avian gammaretrovirus, reticuloendotheliosis virus (REV) (3, 4). In February 2018, a landowner in Gillespie County, TX, reported a Rio Grande wild turkey (Meleagris gallopavo *intermedia*) with pox-like lesions on the nonfeathered areas of the head and neck. A Texas Parks and Wildlife Department veterinarian collected lesion, liver, and blood samples. Histopathology gross findings by the Texas A&M Veterinary Medical Diagnostic Laboratory (College Station, TX) were compatible with the dry and wet forms of FWPV. DNA was extracted by excising 5-mm^2^ sections from a lesion, transferring them into 75 μl HotSHOT alkaline lysis reagent, and further homogenizing them by pestle. The sample was incubated at 95°C for 15 min and then cooled, and 75 μL of HotSHOT neutralization solution was added (5). To determine whether FWPV field isolate TSU-1029 contained an REV proviral genome insertion, PCR using Hot Start *Taq* 2× master mix (NEB; M0496L) and the heterologous primers REV *env* 7F and FPV 203 4R produced an appropriately sized 740-bp product (6), which was sequenced using the Sanger method. A BLASTn (https://blast.ncbi.nlm.nih.gov/) (7) query of the sequence against the nonredundant/nucleotide (nr/nt) database using MegaBLAST produced an alignment to the integration site of a previously sequenced REV-integrated fowlpox virus (GenBank accession number AF006064) (8).

A genomic sequencing library was prepared using KAPA HiFi HotStart ReadyMix (Roche; 07958927001) with the primers TR-1 and TR-2 (9), containing Illumina adapters to amplify the whole REV proviral insertion, flanked by the FWPV open reading frames (ORFs) 201 and 203. The resolved PCR product was excised, tagmented using the Nextera XT DNA library prep kit (Illumina; FC-131-1096), pooled, and sequenced using the MiSeq reagent kit v3 (600 cycles) (Illumina; MS-102-3003). Sequencing produced 4,238 total paired-end reads with a read length of 300 bp. The primer sequences and low-quality and short (<30 bp) reads were trimmed. A consensus sequence was assembled from the reference sequence found under GenBank accession number AF246698.2 (10) and the sequence reads using the CLC Genomics Workbench v6 assemble to reference tool (mismatch cost = 1; indels cost = 2; minimum fraction of similarity between reads and reference = 90%). A total of 92.4% of trimmed reads were mapped (average coverage, 50×). Genome annotation was performed using SeqBuilder Pro v17.2.1 to assign open reading frames and determine the long terminal repeats (LTRs). For analysis of the REV sequence, the annotated reference and consensus genome sequences were aligned using ClustalW in MEGA v11.011 (11), and the FWPV ORF 201 and 203 sequences were manually identified and trimmed. All tools were run with default parameters unless indicated. The proviral REV genome comprises 7,943 bp and has a 52.4% G+C content. BLASTn (nr/nt) searches displayed 7,942/7,943 and 7,937/7,943 nucleotide identities to nearly intact REV proviral genomes within the genomes of FWPV-SD15-670.2 (MH734528) and FWPV-MN00.2 (MH709124) from Merriam’s wild turkeys (Meleagris gallopavo *merriami*) (3). Compared to the full-length REV strain 104865 (KJ756349), this genome shared 7,940/7,943 nucleotide identities with single nucleotide polymorphisms at positions 3 and 7646 and an insertion at position 7. In addition, a 23-bp repeat was deleted at the 5′ end, and the 3′ long terminal repeat was truncated to 222 bp, producing partial LTRs.

### Data availability.

The sequence has been deposited at GenBank under accession number OL857287 and the raw reads under SRA accession number SRX11820217.
